# A Rare Entity of an Unusual Site: Adenomatoid Tumour of the Adrenal Gland: A Case Report and Review of the Literature

**DOI:** 10.4061/2010/702472

**Published:** 2010-06-15

**Authors:** H. El-Daly, P. Rao, F. Palazzo, M. Gudi

**Affiliations:** ^1^Histopathology Department, Hammersmith Hospital, The Imperial College of Science, Technology and Medicine, Du Cane Road, London W120HS, UK; ^2^Department of Surgery, Hammersmith Hospital, The Imperial College of Science, Technology and Medicine, Du Cane Road, London W120HS, UK

## Abstract

This is a case report of a 51 year old male who was found to have an incidental left sided non-functioning adrenal mass on routine medical examination and which was confirmed by CT and MRI scans. A laparoscopic left adrenalectomy was done. On gross examination the tumour was a solitary well circumscribed solid-cystic mass with a homogenous pinkish white cut surface. On microscopic examination, the tumour was composed of variably sized tubules and fenestrated channels lined by bland cuboidal cells to epithelioid cells. There was focal extension to capsule and peri-adrenal fat. Immunohistochemically the tumour cells stained with calretinin, Cam5.2, CK7, vimentin and focally with EMA. Ki-67 fraction was <1%. They were negative for ER, CD31, CD34, Factor 8, chromogranin, synaptophysin S100 and inhibin. A diagnosis of an adenomatoid tumour as made. Adenomatoid tumours are rare benign tumours of mesothelial derivation. The adrenal gland is devoid of a mesothelial lining and the most accepted hypothesis for an adenomatoid tumour originating in the adrenal gland is derivation from mesothelial rests. As the adrenal gland is an extremely rare site of occurrence for an adenomatoid tumour, it is frequently mistaken for adrenocortical tumours or a pheochromocytoma clinically and radiologically.

## 1. Introduction

Adenomatoid tumours are rare benign tumours of the mesothelial origin. The tumour may pose a diagnostic challenge if encountered at unexpected sites, which are devoid of a mesothelial layer. Here we report a case of an adenomatoid tumour of the adrenal gland, clinically suspicious for a malignant tumour.

**Figure 1:**
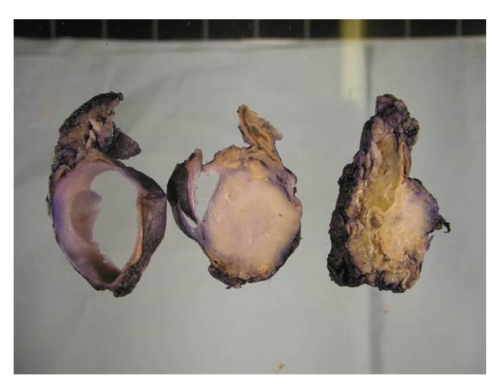
On macroscopy, the tumour has a homogenous solid white cut surface and is associated with a cystic component.

**Figure 2:**
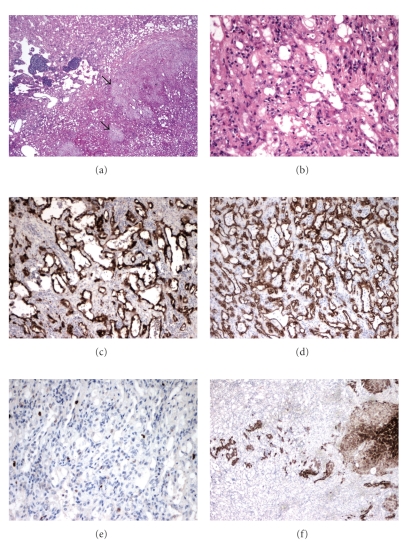
Hematoxylin & eosin (H&E) and Immunohistochemical staining of tumour within adrenal gland. (a) and (b) H&E staining: residual adrenal parenchyma is present in (a) (arrows). (c) Calretinin antibody staining (Leica Microsystems, UK, dilution 1 : 200). (d) Cytokeratin 7 antibody staining (Leica Microsystems, UK, dilution 1 : 100). (e) Ki67 antibody staining (Leica Microsystems, UK, dilution 1 : 50). (f) Inhibin antibody staining (Serotec, UK, dilution 1 : 10).

A 51-year-old male was found to have an incidental left-sided nonfunctioning adrenal mass on routine medical examination. CT and MRI scans revealed solid well-defined left adrenal tumour. Excision was recommended to confirm the nature of the mass, subsequent to which a laparoscopic left adrenalectomy was done. On gross examination the tumour was a solitary well-circumscribed solid-cystic mass with a homogenous pinkish white cut surface (Figure 1). On microscopic examination, the tumour was composed of variably sized tubules and fenestrated channels lined by bland cuboidal cells to epithelioid cells (Figures 2(a) and 2(b)). The tubules surrounded the adrenal cortical tissue, imparting a false infiltrative pattern. Adrenal medulla was barely recognizable. Focal extension to capsule and periadrenal fat was present. Aggregates of mature lymphocytes were seen with foci of calcification. No mitosis, cytological atypia, or nuclear pleomorphism was seen. Immunohistochemically the tumour cells stained with calretinin (Figure 2(c)), Cam5.2, CK7 (Figure 2(d)), vimentin, and focally with EMA. Ki-67 fraction was less than 1% (Figure 2(e)). The tumour cells were negative with ER, CD31, CD34, Factor 8, chromogranin, synaptophysin S100, and inhibin (Figure 2(f)).

Adenomatoid tumours are rare benign tumours of mesothelial derivation, commonly encountered in male and female urogenital tracts. However, other sites such as intestinal mesentery, omentum, pancreas, heart, and pleura have also been documented. To the best of our knowledge <40 cases of adenomatoid tumour of adrenal gland have been reported in the English literature [1–20]. The adrenal gland is devoid of a mesothelial lining, and the most accepted hypothesis for an adenomatoid tumour originating in the adrenal gland is derivation from mesothelial rests. The age of occurrence ranges from 24 to 64 years and has a definite male predilection. The tumour is variably sized, ranging from 0.5 cm to 10.5 cm. The left side appears to be more commonly involved than the right side. The tumour is discovered usually incidentally during workup for unrelated condition; however, occasional cases may present with abdominal pain and hematuria [5, 18]. The tumour is commonly well circumscribed, with occasional ones displaying an infiltrative growth pattern.

The tumour is solid yellow to grey on macroscopy sometimes, associated with a cystic component. Macroscopic calcification is occasionally present. A tubulocystic pattern is seen on histology, lined by epithelioid to flat cells. Signet ring-like cells can be seen [3, 8–10]. Lymphoid follicles with germinal centre formation [4, 5, 9, 10] and dystrophic calcification may be present [3–5, 7], as in our case. Occasional tumours display an infiltrative growth pattern as seen in the present case.

As the adrenal gland is an extremely rare site of occurrence for an adenomatoid tumour, it is frequently mistaken for adrenocortical tumours or a pheochromocytoma clinically and radiologically and hence excised. The tumour can sometimes be seen to extend to the periadrenal tissues, raising the clinical suspicions of an infiltrative process. Signet ring cells, if present, can lead to a misinterpretation of metastatic adenocarcinoma. The commonly encountered tubulocystic pattern with lymphoid follicles can also lead to an erroneous diagnosis of lymphangioma or other vascular neoplasms. However immunohistochemically the tumour is alike to its extra-adrenal counterparts, and diagnosis can be accurately established. Surgical excision is curative, with no known recurrences.

##  Additional Notes

1. Patient consent is not required for this case report because the information are sufficiently anonymised. The figures used include macroscopic image of resection specimen, and pathology slide images do not require consent as they are anonymised by the removal of any identifying marks and are not accompanied by text that could reveal the patient's identity through clinical or personal detail.
2. Competing Interest: “none to declare.”
